# Tef as a case for investment in orphan crop breeding and seed systems development

**DOI:** 10.3389/fpls.2024.1479840

**Published:** 2024-11-20

**Authors:** Zerihun Tadele, Camille Renou, Solomon Chanyalew, Victoria Johnson-Chadwick, George Osure, Mike Robinson, Ian Barker, Dominik Klauser

**Affiliations:** ^1^ Institute of Plant Sciences, University of Bern, Bern, Switzerland; ^2^ Syngenta Foundation for Sustainable Agriculture, Basel, Switzerland; ^3^ Debre Zeit Research Center, Ethiopian Institute of Agricultural Research (EIAR), Bishoftu, Ethiopia; ^4^ Consultative Group on International Agricultural Research (CGIAR), Nairobi, Kenya; ^5^ Botanical Institute, University of Basel, Basel, Switzerland; ^6^ Sustainable Agriculture Initiative Platform, Zurich, Switzerland

**Keywords:** tef, seed system, orphan crops, smallholder - farming sector, sustainable agriculture, neglected crops, Ethiopia

## Abstract

Orphan crops are crops that are of substantial importance to food security and economic growth at a local or regional scale, yet lacking investment in crop improvement and seed systems development. Tef is an example of such an orphan crop. It is vital to economy and food systems in the Horn of Africa, yet investment in breeding and agronomy is very limited. Since almost 20 years, the Syngenta Foundation for Sustainable Agriculture has invested in tef, supporting work to both develop and disseminate improved varieties to farmers in Ethiopia. To date, this has led to the release of four improved varieties. As the project also invested in the development of seed systems for improved tef varieties, it allowed us to monitor seed production and variety adoption over time. The data obtained from seed production monitoring over 7 years and 4 varieties from both formal and informal seed systems shows a total of 1227 tons of tef seed from improved varieties delivered to farmers in Ethiopia. Assuming an average genetic gain of 0.4 tons per hectare, this suggests that the value generated to farmers and local value chains from tef breeding and seed systems development exceeds the investment by an order of at least 2.5. With this paper, we want to make a case for more long-term investment in breeding and seed systems development and stimulate replication of the approach to other orphan crops. We further want to call for a continued investment in tef crop improvement and seed systems development.

## 1 Introduction

Global investment in crop improvement mostly focuses on a few commercially relevant crops. Continuous breeding of these crops over decades has lifted their yield potential substantially (Fischer et al., 2014). In contrast, and especially in developing countries, there are many locally relevant crop species that have been neglected from investment in breeding. Consequently, their genetic potential has yet to be exploited and even minimal investment in crop improvement can lead to substantial yield gains. This, in turn, leads to increasing food security and farmer incomes, especially in the global South (Tadele, 2019). Such under-researched and under-invested crops are commonly referred to as “orphan crops” and include annual and perennial cereals and broad-leaved crops. In addition to their local importance, many orphan crops can help in boosting the productivity and resilience of farming systems through crop diversification and the identification of new disease resistance traits. Genetic improvement and functioning seed systems are a prerequisite for this to materialize.

Tef (*Eragostris tef*) is a cereal crop extensively cultivated in the Horn of Africa. In Ethiopia alone, it is annually cultivated on over three million hectares of land and is the staple food for over 70 million people. Tef is cultivated across diverse agroecological areas with different soils, temperatures, and water conditions. For instance, tef is adapted to semiarid areas with irregular precipitation given its short maturity period of 60-90 days. On the other hand, tef is also popular in high rainfall areas dominated by Vertisol, a heavy clay soil with poor drainage, a condition most other dryland cereals cannot withstand. However, tef seeds can germinate and establish seedlings in waterlogged soils, making it the crop of choice on Vertisols which constitute about 10% of arable land in Ethiopia (Chanyalew et al., 2019).

Apart from its desirable agronomic traits, tef also has nutritional benefits: The tiny grains are packed with essential nutrients, such as calcium and iron, and rich in vitamins given that during processing the bran and germ retain on the kernel. Further, tef straw is considered a high-value side product given its palatability and nutritional properties as a livestock feed (Shumoy and Raes, 2017). Internationally, the fact that tef is a gluten-free cereal and has a low glycemic index has recently led to it being branded as a “super food” and being included in various lifestyle products (Provost, 2014). Further, the appetite of a growing Ethiopian diaspora for injera, the flat bread made of tef which is part of the region’s culinary and cultural heritage, has also increased demand from export markets (Lee, 2018). As a result of growing demand, the area under tef cultivation in Ethiopia has increased by 59% in the last 25 years. The combination of high market prices and low production risks keep on encouraging many farmers to move to tef cultivation (Chanyalew et al., 2019).

Despite of the growing demand for tef and its importance as a staple crop in a yet food insecure part of the world, tef has neither attracted substantial public nor private sector investment in breeding or seed sector development. As a result, currently used cultivars are nowhere near the crop’s yield potential and major production and resilience challenges, such as lodging, have yet to be targeted by crop improvement. It follows that even limited investment in crop improvement can have a significant positive effect on the crop’s productivity. For instance, limited government investment in breeding and seed systems has increased the crop’s productivity by 157% in the last 30 years, from 0.7 t/ha in 1994 to 1.8 t/ha in 2020. However, comparing average tef yield to maize (up to 4.2 t/ha) and wheat (up to 3.1 t/ha) in Ethiopia shows the low productivity of tef to date (Chanyalew et al., 2019).

In response to the underinvestment in tef breeding and seed systems and its importance as a staple crop in the Horn of Africa, the Syngenta Foundation for Sustainable Agriculture and the University of Bern entered a partnership in 2006 to develop improved tef varieties through state-of-the-art, non-transgenic breeding techniques. This partnership was later joined by the Ethiopian Institute for Agricultural Research (EIAR), the Swiss Office for Development and Cooperation (SDC), as well as small-scale private seed companies in Ethiopia to ensure the release and delivery of improved tef cultivars in the country.

## 2 Methodology and results

The project’s implementation plan followed a sequenced approach for developing improved genetics and delivering them to farmers. This included trait identification, trait introgression in local elite varieties, testing and release, and finally delivery through formal and informal distribution channels. This strategy was discussed in detail by Cannarozzi et al. (Cannarozzi et al., 2018).

The project’s evolution was supported by a long-term resource plan from 2006-2023, which gradually shifted resource allocation from trait identification to breeding, release and eventually seed production and delivery to ensure benefits from improved varieties materialize in farmers’ fields. Over the 18 years of the project to date, a total of 8.5 million CHF (10 million USD) has been deployed to support tef breeding and seed systems development.

### 2.1 Breeding

Focusing on the major abiotic constraints to tef production and with support of academic and private partners across Europe and Africa, the project used a large-scale phenotyping and genotyping approach to screen for genetic material with improved tolerance to lodging, drought and soil acidity. This led to the identification and characterization of novel genetic variants with semi-dwarf stature and lodging-tolerance properties (Jöst et al., 2015; Plaza-Wüthrich et al., 2016), acid soil tolerance properties (Desta et al., 2017; Abate et al., 2022), mechanical properties for lodging tolerance (Blösch et al., 2020), enhanced drought-tolerance and altered stomata structure (Blösch et al., 2019, Kebede et al., 2022).

After screenings for traits of interest by the University of Bern, candidate lines were shipped to Ethiopia for crossing with high-yielding, locally adapted cultivars. To date, this has led to the establishment of over 100 crosses that showed desired traits, which have subsequently been thoroughly assessed in field trials across Ethiopia. For this, the project engaged with EIAR in crossing, trialing, and releasing improved varieties to farmers. To assess the varieties under on-farm conditions across diverse agro-ecological zones, recombinant inbred lines from the crosses have been evaluated in up to 34 growing regions in Ethiopia (Cannarozzi et al., 2018). After thorough testing, Tesfa, the first variety with enhanced lodging resistance was approved for release in 2017 (Kebede et al., 2018). This was then followed by Bora (in 2019) and Boni (in 2021), the two cultivars with improved drought tolerance and Ebba (in 2019), a high-yielding variety in high moisture areas (**Table 1**).

**Table 1:** Varieties released in Ethiopia and year, traits, and average yields from on-farm trials across 34 regions across Ethiopia.

Variety	Year of Release	Traits	Average on-farm yield (t/ha)
Tesfa	2017	Lodging tolerance	2.0-2.4
Ebba	2019	High yield	2.0-2.6
Bora	2019	Drought tolerance	1.8-2.4
Boni	2021	Drought tolerance	2.0-2.6
Bishoftu	2021	Early maturity	2.0-2.8
Kulle	2022	Late maturity, high yield	2.4-3.0
Bereket	2022	Late maturity, high yield	2.4-3.2

On-farm trials of all these varieties are being continuously conducted to understand farmer preferences and estimate potential yield gains over currently used genetics (Bekele et al., 2017, 2019, 2022). In general, all varieties released by this project were shown to increase on-farm yields of lead farmers by about 0.2 – 0.8 t/ha, compared to local check varieties (**Table 1**).

### 2.2 Dissemination

The dissemination of these varieties was first initiated through lead farmers in Central districts of Ethiopia. This strategy proved to be successful to deliver improved varieties through informal systems and to facilitate farmer to farmer peer learning through field days and other activities (Bekele et al., 2019). However, to deliver genetic gain to other regions of Ethiopia another strategy was required that allowed for large-scale seed production and logistics. In addition to investing in informal seed delivery channels, the project invested in formal seed delivery systems to increase the speed of delivery of improved genetics in 2018. This led to a partnership with two local seed companies that have since produced and delivered seeds of improved varieties according to international quality and purity standards whilst also keeping records of volumes of seed produced by variety and year. These seed companies then delivered the produced seed to regional, informal seed multiplication centers that further multiplied the seed and delivered to farmers.

### 2.3 Impact

Informal seed channels, such as farm-saved seed or farmer-to-farmer seed exchange represent the predominant seed delivery system for improved genetics and new varieties of most orphan crops, including tef. However, through such systems, diffusion can be slow and tracking the dissemination of new varieties difficult. On the other hand, the reach of formal seed systems can be more readily estimated as seed production volumes and sales are recorded by seed companies. The hereby presented project supported both informal and formal delivery channels recorded production for both channels. Production volumes for all supported varieties by year are shown in **Table 2** for formal seed delivery and **Table 3** for informal seed delivery systems. These data were provided by the seed companies that the project supported (formal channels) and the volume of seed produced by EIAR to be delivered to lead farmers (informal channels). It is important to note that there is limited control over these delivery channels beyond these early steps of seed multiplication. Deriving from recorded seed production data, observed on-farm yield gains from multilocation trials, and planting rates we estimated the value creation by using SFSA’s seed for impact model (www.seeds2b.org). This model basically uses the following formula: (Volume of seed produced (tons)/average planting rate (tons/ha) X expected on farm yield gain (t/ha) X farm-gate price for crop produced (USD/t). The model does neither include any additional production costs that result from increased production, nor any additional effects from on-farm seed multiplication or saving seed for consecutive seasons, which both common for tef farming systems in Ethiopia (Hauenstein, 2015). Records for seed production per variety and year since 2017 are featured in **Table 2** (formal seed sector) and **Table 3** (informal seed sector) below.

**Table 2:** Tons of seed produced through formal seed sector per variety and year.

Variety	2017	2018	2019	2020	2021	2022	2023	Total Volume of Seed Produced (tons)
Tesfa	1	5	6	26	12	4	2	56
Ebba				6	21	35	65	127
Bora				6	11	21	38	76
Boni					5	18	34	57

**Table 3:** Tons of seed produced through informal seed sector per variety and year.

Variety	2017	2018	2019	2020	2021	2022	2023	Total Volume of Seed Produced (tons)
Tesfa			12	37	14	3		66
Ebba				7	98	183	342	630
Bora						35	58	93
Boni					2	45	75	122

From the recorded seed production volumes we calculated hectares planted with improved varieties, using an estimated planting rate of 14kg of tef seed per hectare, which is within the recommended planting rate of 10-15 kg/ha. We then used this estimate of planted hectares to calculate improved production for new varieties, using observed yield-gains from multi-location trials (**Table 4**). As an average, we used an estimated yield gain of 0.4 tons of tef grain per hectare for improved varieties and based on the yield data we reported in **Table 1** from multilocation trials across Ethiopia. This is a conservative estimate, taking in account our observation that average yields for improved varieties tend to be slightly below initial reports from on-farm trials. Further, it is based on farmers not changing any other aspects of growing the crop.

**Table 4:** Acreage planted and estimated surplus value generated through improved Tef varieties released by the project.

Variety	Seed production 2017-2023 (tons)	Area planted (hectares)	Value of surplus production (million USD)
Tesfa	122	8700	2.1
Ebba	757	54000	13.0
Bora	169	12100	2.9
Boni	179	12700	3.1
**Total**	**1227**	**87500**	**21.1**

Assuming a farm-gate price of 0.6 USD per kg of tef grain, we derived an additional value created at 21 million USD to date. This notably without the need for additional inputs or changes in agronomic practices, as yield gains are purely derived from genetic gain (**Table 1**). With an overall project investment of 8.5 million CHF, this equals a social return on investment (SROI) of 2.5 USD per 1 USD invested in the project to date. The hereby calculated SROI does not include the effect of local seed multiplication, which has not been monitored by this project. It can be assumed that, given the prevalence of local, informal seed delivery channels, the reach of these improved varieties to be at least an order of magnitude higher. The SROI is expected to further increase in the future with the adoption of new varieties continuing to grow for more years to come.

## 3 Way forward

The hereby presented project suggests a SROI of investment in orphan crop breeding and seed systems development to be at least 2.5 in the case of tef in Ethiopia. This is aligned with estimates for the SROI for investment in breeding and seed delivery systems of other smallholder-relevant cereal crops, such as Sorghum and Millet (Alston et al., 2021). It further underlines the need to ensure that investment in breeding is accompanied by investment in seed delivery mechanisms to ensure that genetic gain is created, but also delivered to farmers to materialize and ensure adoption and impact (Woltering et al., 2019). Creating both the technology, as well as sustainable and scalable delivery mechanisms is essential to reach beneficiaries, which was also observed for other orphan crops.

Such investments to create both the technology and ensure its sustained delivery to farmers are likely to require longer-term, programmatic funding cycles that allow for stage-gate approaches from development to release to production and adoption. This is also needed to overcome inherent bottlenecks in delivering improved crop varieties to smallholder farmers, such as balancing and buffering seed supply and demand in the light of fluctuating markets and farmer preferences for farm-saved seed (Barker et al., 2020). The hereby presented project is not an exception, with a continued, and continuing, investment of 18 years to date. Sadly, this stands against prevailing trends towards more short-term, project-based investment approaches that many donors prioritize today and that has a limited track record of leading to sustained adoption of innovation and therefore impact (Schut et al., 2020).

There are various additional agronomic and seed-related constraints that need to be tackled for tef. They include better planting technologies to improve crop establishment and crop yields, the inclusion of new, consumer and processor-relevant breeding traits to build more resilient tef value chain, and, importantly, a better understanding of protection mechanisms for genetic diversity (Cannarozzi et al., 2018). The latter also to avoid potential intellectual property conflicts as the crop gets more relevant in other parts of the world, such as the United States, Southern Europe, South Africa, and Australia (Abraham, 2015).

Whereas investment in increasing the productivity of agricultural systems is vital to create value for farmers in developing countries, it is important to bear in mind that most of the value creation – and jobs – from agricultural value chains come from activities downstream of production, such as processing and retail (Cucangna and Goldsmith, 2017). We therefore see it as a natural progression of this project moving forward to engage with tef processors and retail and to serve continuously growing domestic and international markets for products that create value and employment opportunities locally.

We also hope that by sharing the results of this project, we can demonstrate the positive SROI of long-term investment in orphan crop breeding and seed systems development and can catalyze further interest and investment in similar approaches for Tef and other neglected crops.

## Data Availability

The original contributions presented in the study are included in the article/supplementary material, further inquiries can be directed to the corresponding author/s.
